# A genome-wide association study of cognitive impairment in a Chinese cohort of 1,040 individuals

**DOI:** 10.1007/s00439-026-02887-6

**Published:** 2026-10-17

**Authors:** Maggie Haitian Wang, Yexian Zhang, Rui Sun, Fudong Li, Tao Zhang, Xue Gu, Jingzhi Lou, Lirong Cao, Shimin Su, Hong Zheng, Caiqi Liu, Marc Ka Chun Chong, William Ka Kei Wu, Jun Yu, Benny Chung-Ying Zee, Junfen Lin

**Affiliations:** 1 Center for Clinical Research and Biostatistics, JC School of Public Health and Primary Care (JCSPHPC), The Chinese University of Hong Kong (CUHK), Hong Kong SAR, China; 2 Zhejiang Provincial Center for Disease Control and Prevention, 3399 Binsheng Road, Hangzhou, 310051 China; 3 CUHK Shenzhen Research Institute, No. 10 Yuexing 2nd Road, Shenzhen, 518172 China; 4 Beth Bioinformatics Co. Ltd, Hong Kong SAR, China; 5 School of Medicine, Warshel Institute for Computational Biology, The Chinese University of Hong Kong (CUHK-SZ), 2001 Longxiang Road, Shenzhen, 518172 China; 6 Department of Anaesthesia and Intensive Care, CUHK, Hong Kong SAR, China; 7 Li Ka Shing Institute of Health Sciences, CUHK, Hong Kong SAR, China; 8 State Key Laboratory of Digestive Diseases, Institute of Digestive Diseases and Department of Medicine and Therapeutics, CUHK, Hong Kong SAR, China

## Abstract

While genetic heritability plays a major role in the development of cognitive impairment, most genetic risk factors are identified from studies of European populations. We conducted a genome-wide association study (GWAS) on cognitive impairment in the Chinese population by sequencing 1,040 matched case-control individuals from a longitudinal cohort of Zhejiang Province, China. We detected 19 SNPs clustered in 15 genomic loci reaching suggestive significance (*P* < 1 × 10^− 5^). Eleven of these genomic loci are newly associated with Alzheimer’s disease (AD) or cognitive impairment, among which five (*GRP39*, *FAT1*, *EDIL3*, *ZNF596*, and *PDGFD*) show nominal evidence of replication in both external GWAS of AD in East-Asian populations and summary statistics of AD in diverse populations. The detected loci are implicated in micronutrient homeostasis including zinc (*GPR39*), iron (*STEAP1*), potassium (*KCNJ6*), and vitamin *K* (*VKORC1L1*), cell connections (*EVPLL*) through cadherin regulations (*PCDH18*, *FAT1*), regulating amyloid-beta precursor protein (APP) (*EDIL3*), formation of amyloid-β (*EDEM1*) and neurofibrillary tangles (*PDGFD*, *ZNF596*), neuronal growth (*NEGR1*), and phosphatase regulations (*SETBP1*, *TPTE*). This study presents a robust GWAS for cognitive impairment in the Chinese population and highlights a promising set of therapeutic targets for neurodegenerative disorder.

**Supplementary Information:**

The online version contains supplementary material available at https://doi.org/10.1007/s00439-026-02887-6.

## Introduction

Cognitive impairment, characterized by cognitive deficits and spans a broad clinical spectrum, ranging from mild cognitive impairment (MCI) to dementia (Mian et al. 2024; Taylor-Rowan et al. 2022). Declining cognitive function is associated with reduced ability to live independently. The prevalence of cognitive impairment in the general elderly population ranges from 5.1% to 41% (Pais et al. 2020). More than half of individuals with MCI would progress to dementia within five years (Gauthier et al. 2006), among whom about 45% will develop Alzheimer’s disease (AD) (Blennow et al. 2006; Gatz et al. 2006; Solfrizzi et al. 2004). The etiology of dementia is influenced by both polygenic effects and environmental risk factors including age, education attainment level, and social engagement (Bellou et al. 2017). The largest twin study to date estimated that genetic factors account for 60–80% of the risk of AD (Gatz et al. 2006). The most prominent genetic risk factor is the *APOE* gene (Gauthier et al. 2006; Petersen et al. 1995), with the ε4/ε4 genotype increasing the risk of late-onset AD and dementia by 5-fold and 2-fold, respectively, compared to the wild-type (ε3/ε3) in the European populations (van der Lee et al. 2018a, b). Beyond *APOE*, large scale genome-wide association studies (GWAS) and meta-analyses identified near 75 risk variants associated with AD (Bellenguez et al. 2022; Jansen et al. 2019; Lambert et al. 2013; Marioni et al. 2018; Wightman et al. 2021). These genes are involved in immune system function (*CLU*, *CR1*, *CD33*) (Crehan et al. 2012; Foster et al. 2019; Jiang et al. 2014), endocytosis pathways (*BIN1*, *PICALM*) (Tan et al. 2013b; Xu et al. 2015), and lipid processing pathways (*APOE*, *CLU*), among others (Foster et al. 2019; Serrano-Pozo et al. 2021). In China, the prevalence is estimated at 22.24% for cognitive impairment,12–16% for the MCI, 6.0% for dementia, and 3.9% for AD in the elderly population (Jia et al. 2020; Lu et al. 2021; Qin et al. 2022; Xue et al. 2018). However, only a limited number of genome-wide association studies (GWAS) have been conducted on AD or dementia in Chinese population. Zhou et al. (2018), using a cohort of 489 AD, 260 MCI, and 223 controls, identified variants with suggestive significance in the *GCH1* and *KCNJ15* genes, which are involved in hydrolysis of guanosine triphosphate and potassium channel function, respectively (Zhou et al. 2018). Jia et al. (2020) identified the *RHOBTB3* that enables ATP binding activity and retrograde transport and the *CHODL*, a type I membrane protein involved in neurite outgrowth and elongation (Sleigh et al. 2014), from a two-stage GWAS cohort involving 3,913 AD and 7,593 controls (Jia et al. 2021). Another study based on 139 twin pairs identified a long non-coding RNA (lncRNA) with suggestive significance, although its biological link to cognitive impairment is unclear (Xu et al. 2017). Notably, candidate genes for AD found in European populations often show only modest association in Chinese populations (Jiao et al. 2015; Tan et al. 2013a; Wang et al. 2012, 2016). Therefore, the genetic architecture underlying cognitive function in the Chinese population remains largely unexplored.

To address this gap, we conducted a GWAS of cognitive impairment in a Han Chinese cohort comprising 1,040 subjects, utilizing low-depth whole genome sequencing and *APOE* genotyping. Following the identification of associated genetic signals, we performed replication studies using two East-Asian GWAS datasets of the UK Biobank and a Japanese cohort. This was followed by polygenic risk score and functional analysis of the detected single nucleotide polymorphisms (SNPs). Remarkably, the detected loci exhibit highly relevant biological roles in the etiology of brain disorder, many of which are reported for the first time in the context of dementia or AD.

## Materials and methods

### Study population and sampling design

The participants in this study were recruited from a longitudinal cohort of over 10,000 individuals enrolled in the Zhejiang Major Public Health Surveillance Program, managed by the Zhejiang Provincial Center for Disease Control and Prevention (Zhejiang CDC). Between 2014 and 2016, these participants were surveyed annually to assess their health status, medical history, and screened for dementia by the Mini-Mental State Examination (MMSE) (Zhang et al. 2018). The inclusion criteria of participants were being of Han-Chinese, permanent residents aged 60 or older, and having lived in the surveyed county for at least six months each year during the survey period.

Cognitively impaired cases were identified based on education-adjusted MMSE cutoff scores for cognitive impairment specific to the Chinese population (< 17 for illiteracy, < 20 for primary education, and < 24 for secondary education or higher) (Qin et al. 2022; Zhang et al. 1990). Additionally, participants had to meet the MMSE cutoff scores in both of the last two years of the longitudinal survey. For the control group, a stratified random sampling method was used to control for major environmental risk factors associated with dementia and AD (Kunkle et al. 2019; Lautenschlager et al. 2008; Ott et al. 1995). Specifically, for each case, two control samples were randomly selected who did not meet the MMSE cutoffs during the survey period, matched by education-level, gender, and age (within two years of the case subject). This process resulted in a total number of 1,040 participants, including 377 cases and 663 controls for the genetic sequencing study. Written informed consent was obtained from all participants, and ethical approval was granted by the Ethics Committee of Zhejiang CDC and the Joint Chinese University of Hong Kong–New Territories East Cluster Clinical Research Ethics Committee.

### Whole genome sequencing (WGS) and genotyping of *APOE* SNPs

Low-pass whole-genome sequencing was conducted on the Illumina Hiseq X Ten platform (Illumina, Inc., San Diego, CA, USA) at an average depth of 5-fold. Variant calling was carried out using Sentieon DNAseq, mapped to the human reference genome (GRCh37), resulting a total number of 61,522,284 variants. The *APOE* variants rs429358 and rs7412 were separately genotyped by polymerase chain reaction (PCR) on the GeneAmp™ PCR System 9700 (Applied Biosystems, Foster City, CA, USA), followed by Sanger sequencing on the 3730XL DNA Analyzer (Applied Biosystems).

### Quality control (QC) and imputation

The quality control (QC) of the genotyped data followed pre-established protocols (Anderson et al. 2010). QC of the sequencing data begun by excluding individuals with discordant sex information, a call rate < 0.95, or elevated heterozygosity rates. While we do not have the family relationship information of the enrolled subjects, identity-by-descent (IBD) analysis detected three pairs with IBD proportion in the range of 0.4–0.6, suggesting first-degree relatives (parent-offspring or full siblings), and no pair shows value typical of second-degree relatives (~ 0.25), based on genome-wide identity-by-state (IBS) data performed using PLINK with the “--genome” parameter. Therefore, one individual from each related pair was removed where the estimated IBD proportion > 0.1875. Subsequently, we filtered out variants with a missing value percentage > 0.05, a minor allele frequency (MAF) < 0.01, a Hardy–Weinberg Equilibrium (HWE) *p*-value < 1 × 10^− 7^, or a significant difference in genotype call rates between cases and controls (*p*-value < 1 × 10^− 5^). After these steps, the dataset consisted of 1,020 individuals, including 370 cases and 650 controls, and 1,859,965 SNPs. For imputation, the genotype data were phased by Eagle2 (Browning and Browning 2011) and imputed with minimac3 (Das et al. 2016) on the Michigan Imputation Server (https://imputationserver.sph.umich.edu), using the 1000 Genomes phase 3 reference panel (Auton et al. 2015) for the East Asian population. The imputation resulted in 27,044,035 variants. Post-imputation variants with an imputation quality score R^2^ < 0.3 or a MAF < 0.01 were removed, resulting in 5,722,509 variants. The analyses were conducted using PLINK 1.9 (Purcell et al. 2007) and R (version 3.6.3) (R Core Team 2013).

### Power estimation

Power estimations assume an additive disease model with significance threshold 1 × 10^− 5^, conducted using code provided by Matti Pirinen from the University of Helsinki (https://www.mv.helsinki.fi/home/mjxpirin/GWAS_course/material/GWAS3.html).

### Statistical analysis for association analysis

Genome-wide association testing was performed using a generalized linear mixed model (GLMM) implemented in the R package GMMAT (version 1.4.0) (Chen et al. 2016). SNPs, age, gender, education level, smoking status, and drinking status were treated as fixed factors, while the standardized empirical genetic relationship was included as a random factor to account for potential population structure (Chen et al. 2016; Zhou and Stephens 2012, 2014). Effect sizes were estimated using the Wald test in GMMAT. Manhattan plot and quantile-quantile (Q-Q) plot were generated using the R package qqman (version 0.1.8) (Turner 2014). The linkage disequilibrium score regression (LDSC) intercept was calculated to assess the potential inflation of type I error rate, using in-sample estimates of the LD structure, which were obtained by applying LDSC’s command “--l2” with the usual 1-centimorgan window (--ld-wind-cm 1) (Bulik-Sullivan et al. 2015). We defined a locus as a region with 500-kb window flanking a SNP with *P* < 1 × 10^− 5^ and merged overlapping regions. To identify independent associations within the detected loci, conditional analysis of each regional variant was performed using the GMMAT, with a 1-Mb window centered on the most significant (lead) SNP and including it as a covariate. Regional association signals surrounding each independent lead SNP were plotted by LocusZoom (http://locuszoom.org/) (Pruim et al. 2010) and 1000 Genomes East Asian reference data (Auton et al. 2015). The association between *APOE* genotypes and the cognitive impairment outcome was assessed using the Wald test in GMMAT, adjusting for age, gender, education level, smoking status, drinking status, and population structure with the standardized empirical genetic relationship matrix.

### Inference of genetic ancestry

To identify potential ancestry outliers, we merged our genotype data with 1000 Genomes Phase 3 data (Auton et al. 2015), retaining only the SNPs shared between the two datasets. The 1000 Genomes Phase 3 reference panel includes 2,504 samples, which represents five continental ancestry populations: African (AFR, *n* = 661), American (AMR, *n* = 347), East Asian (EAS, *n* = 504), European (EUR, *n* = 503), and South Asian (SAS, *n* = 489). These SNPs were then pruned using PLINK’s command “--indep-pairwise 500 50 0.05”, resulting 61,325 pruned variants. We then used the principal components analysis (PCA) by PLINK --pca to assess population structure and detect potential outlying subjects.

### Heritability analysis

The SNP-based heritability ($\:{h}_{g}^{2}$) was estimated using the genomic restricted maximum likelihood (GREML) method implemented in the GCTA software with the “--reml” flag (Yang et al. 2011). The genetic relationship matrix (GRM) between pairs of individuals was estimated from the SNPs with MAF > 0.01 using the command “--make-grm --maf 0.01”. The heritability estimated on the observed scale was transformed to the liability scale using a population prevalence of 0.05 (Wightman et al. 2021) and the prevalence of cognitive impairment in our sample (370 out of 1020 individuals).

### Replication data and meta-analysis

For replication study, we utilized publicly available AD GWAS summary statistics from European (Kunkle et al. 2019; Mukherjee et al. 2020), African American (AFR) (Kunkle et al. 2021), and Japanese populations (Shigemizu et al. 2021). Additionally, we performed GWAS analysis on proxy AD across four ancestry groups in the UK Biobank.

We performed the East Asian-specific meta-analysis by combining the results of: (1) SNPs from our imputed GWAS data on cognitive impairment, (2) AD summary statistics of the UK Biobank (UKBB) East Asian (EAS) population, calculated in house using the proxy AD as phenotype; and (3) a published AD GWAS in the Japanese population (Shigemizu et al. 2021), through the inverse-variance fixed-effect method in the METAL software (Willer et al. 2010). Heterogeneity among these studies were assessed by Cochran’s Q test and Higgins’s I2 test statistic. East Asian-specific meta-analysis were conducted at the locus (genome region) level to identify loci that may share association with cognitive impairment and AD. For each locus, we tested all variants within 500 kb of the lead SNP and selected the most strongly associated variant (Bellenguez et al. 2022; Keaton et al. 2024). We report the genomic loci reaching the nominal significant threshold (*p*-value < 0.05) and Bonferroni corrected significant threshold (*p*-value < 0.05/15 = 3.3 × 10^− 3^) in the meta-analysis and had the same direction of effect across all primary GWAS analysis.

For the 15 independent lead variants associated with cognitive impairment, we examined their replications in AD GWAS across multiple populations. If the lead SNP was not observed in the replication GWAS, we searched for a proxy SNP in high LD (*r*^2^ > 0.6 in the 1000 Genomes EAS panel) as a substitute using the R package LdlinkR (version 1.4) and selected the proxy SNP with the most significant association in the replication GWAS (Keaton et al. 2024).

*Data 1.* UK Biobank (UKBB). The UKBB is a prospective cohort study that enrolled over 500,000 participants aged 40–69 years from the United Kingdom from 2006 to 2010 (Bycroft et al. 2018). The genetic data of UKBB includes 96 million variants for 488,377 participants, with self-reported ancestry breakdowns of 94% Europeans, 2% West or Southeast Asians, and 2% African (Khan et al. 2022). The ancestry groups of UK Biobank participants were estimated with the PCA approach (Privé et al. 2022). Specifically, the UKBB participants were projected onto principal components derived from the 1000 Genomes Project reference panel. Participants were then assigned to one of four super-populations (EUR, AFR, EAS, or SAS) based on their Euclidean distance to the reference population centers in the PCA space. The QC procedures were performed on each population independently; imputed SNPs with MAF < 0.001, HWE *p*-value < 1 × 10^− 7^ or imputation INFO score < 0.3 were excluded. We exclude individuals with sex chromosome aneuploidy, mismatches between the self-reported sex and the genetically inferred sex, outliers based on heterozygosity and missingness, or those with ten or more putative third-degree relatives (Marioni et al. 2018). According to the AD-by-proxy phenotype definition used in Jansen et al. (Jansen et al. 2019), a proxy AD phenotype was constructed for participants based on their own AD status, as well as the AD statuses and ages of their parents. Association analyses were conducted separately for each population using logistic regression models adjusted for sex, age, and first 10 PCs, implemented with PLINK software (Purcell et al. 2007).

*Data 2*. Japanese cohort. Summary statistics from a Japanese GWAS cohort were obtained from the National Bioscience Database Center (NBDC) Human Database (Shigemizu et al. 2021). The dataset comprises 3,962 AD cases, 4,074 controls, and 4,852,957 variants.

*Data 3*. Stage 1 of the International Genomics of Alzheimer’s Disease Project (IGAP). The IGAP Stage1 dataset includes 21,982 AD cases, 41,944 cognitively normal controls, and 11,480,632 variants of European ancestry (Kunkle et al. 2019).

*Data 4*. African cohort. This dataset represents the largest publicly available AD GWAS from the African population (2,748 cases and 5,222 controls), obtained from the NIAGADS dataset under accession number NG00100 (Kunkle et al. 2021).

*Data 5*. Cognitively defined late-onset AD subgroups. The summary statistics for cognitively defined late-onset AD subgroups were obtained from the National Institute on Aging Alzheimer’s Disease Data Storage Site (NIAGADS) project NG00073 (Mukherjee et al. 2020). This dataset combined genetic information from five consortia: the Adult Changes in Thought (ACT) study, the Alzheimer’s Disease Neuroimaging Initiative (ADNI), the Religious Orders Study (ROS), the Memory and Aging Project (MAP), and the University of Pittsburgh (PITT) study. The subgroups were categorized based on cognitive performance in memory, executive functioning, visuospatial functioning, and language at the time of AD diagnosis. The subgroups include: Language predominant AD subgroup (Cases: 324, Controls: 3,444), Memory predominant AD subgroup (Cases: 653, Controls: 3,444), Multiple substantial relative impairment AD subgroup (Cases: 144, Controls: 3,444), No domain with substantial relative impairment AD subgroup (Cases: 974, Controls: 3,444), and Visuospatial predominant AD subgroup (Cases: 141, Controls: 3,444).

### Polygenic risk score (PRS) analysis

To evaluate the amount of variation in cognitive impairment explained by the detected SNPs, we constructed PRS using the SNPs that reached suggestive significance (*P* < 1 × 10^− 5^), calculated as the sum of an individual’s risk alleles weighted by the effect sizes of risk alleles. The PRS was generated using the “--score” option in PLINK 1.9 (Purcell et al. 2007) and subsequently normalized. To evaluate the performance of the PRS, we calculated Nagelkerke’s pseudo *R*^*2*^ and generated plots showing ORs and confidence intervals (CIs). The *R*^*2*^ was estimated by comparing a logistic regression model that includes the PRS, age, gender, education level, smoking status, drinking status, and the first 10 PCs for population structure, to a reduced model containing only the covariates. To estimate the ORs and CIs across PRS deciles, individuals were divided into ten deciles based on their PRS distribution, with 1 representing the lowest and 10 the highest. The OR for each PRS decile group is calculated relative to the lowest decile using a logistic regression model (Grove et al. 2019; Keaton et al. 2024).

### Functional annotation

Variants were mapped to the nearest protein-coding gene in the human reference assembly (GRCh37/hg19). For each risk locus, the closest protein-coding gene to the lead SNP is considered the most probable candidate. To assess whether the detected SNPs are enriched in specific brain regions, we applied the LD score regression for specifically expressed genes (LDSC-SEG) using 13 GTEx brain region annotations (Finucane et al. 2018) and the Bonferroni-corrected significance level was 0.05/13 = 3.8 × 10^− 3^.

We conducted gene-based association analysis and pathway (gene-set) enrichment analysis using MAGMA (de Leeuw et al. 2015), implemented in the FUMA platform (Watanabe et al. 2017). The gene-based signals were assessed by their association to tissue specific gene expressions of 30 general tissue types from GTEx V8, while the pathway enrichment was evaluated by comparing whether genes within a certain pathway were more strongly associated with the phenotype compared to other genes, based on 17,004 gene sets (6,488 curated gene sets and 10,516 gene ontology terms) from the MSigDB database (de Leeuw et al. 2016; Liberzon et al. 2015; Subramanian et al. 2005). The Bonferroni-corrected significance thresholds were set at 0.05/30 = 1.7 × 10^− 3^ for GTEx tissues and at 0.05/17,004 = 2.9 × 10^− 6^ for gene set analysis.

## Results

### Inferences from Zhejiang GWAS of cognitive impairment

We recruited 377 cases and 663 controls matched by age, gender and education level in a 1:1 or 1:2 ratio from a longitudinal cohort in Zhejiang Province, China (Methods). An individual is classified as a case with cognitive impairment if his MMSE score exceeds the Education-adjusted cutoffs for cognitive impairment in the Chinese population (Zhang et al. 2018) in both the second and third years of a three-year follow-up period. The MMSE score distribution among cases is shown in Fig S1. The mean age of participants at baseline was 76.3 years (SD 6.9) with 59.3% being female. After quality control, data of 1,020 subjects were retained, including 370 cases, 650 controls and 61.5 million variants. No population stratification was detected, as indicated by the LDSC intercept 0.96 (SE 0.0026), genomic inflation factor λ 1.01, and the Q-Q plot (Fig S2). Principal component analysis result confirmed that all subjects belonged to the East Asian population (Fig S3, Methods). SNP-heritability estimated by the GCTA software (Yang et al. 2011) was 0.59 (SE 0.3, *P* = 0.01) based on a population prevalence of 0.05 (Wightman et al. 2021).

**Fig. 1:**
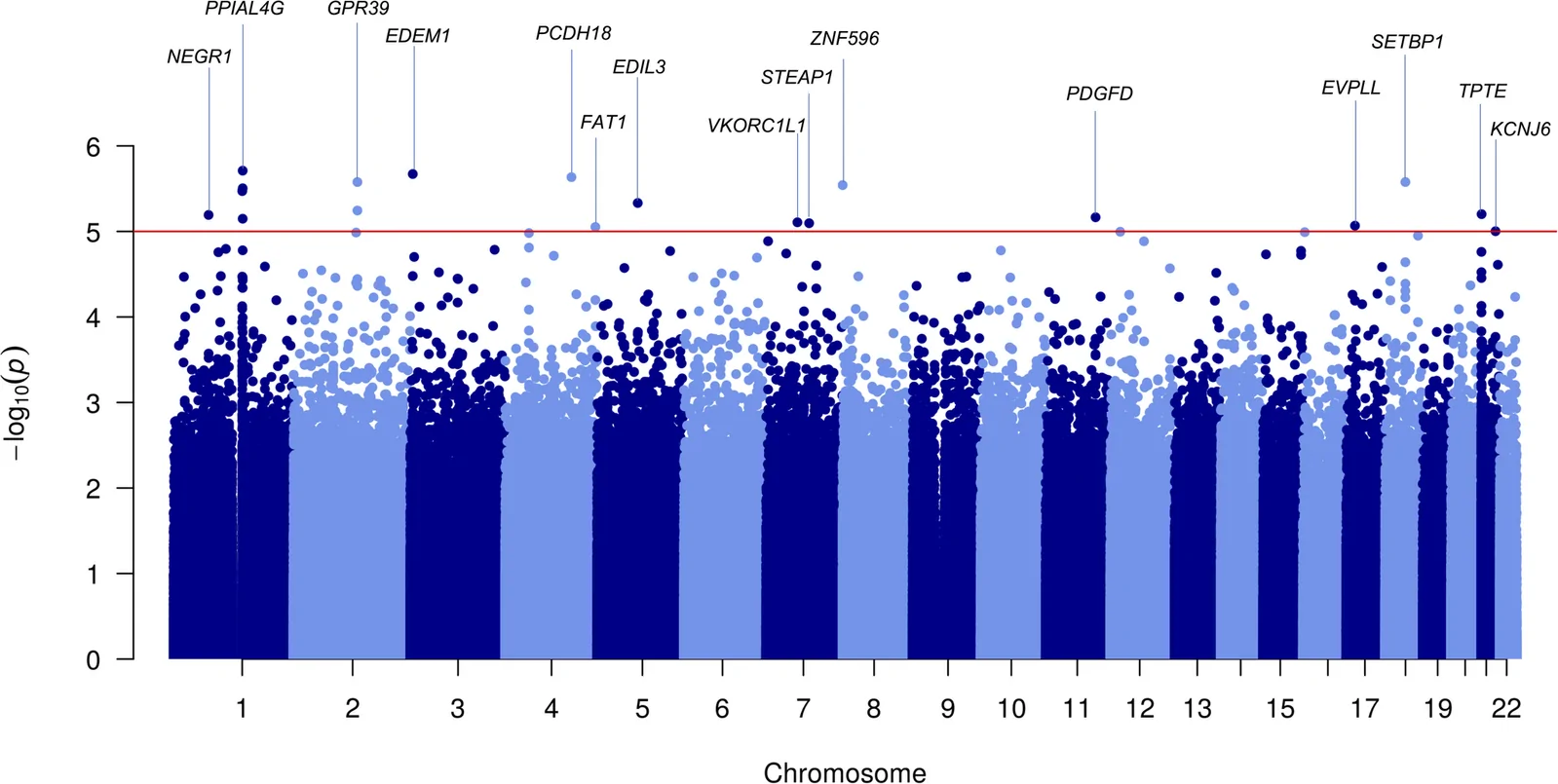
Genome-wide association study Manhattan plot for cognitive impairment in the Zhejiang Population. Nineteen variants distributed in 15 genomic loci reached suggestive significance threshold (*P* < 1 × 10^− 5^, red line) in this GWAS of cognitive impairment in Chinese population

We employed a generalized linear mixed model controlling for covariates to detect genetic association signals in the Zhejiang GWAS data. The Manhattan plot of the genome-wide association signal is placed in Fig. 1. Analysis revealed that a total number of 19 variants reached a suggestive level of significance (*P* < 1 × 10^− 5^), clustered within 15 independent genomic loci (Table 1, Table S1). All of these 15 loci are novel to the confirmed list of AD genes compiled by the Alzheimer’s Disease Sequencing Project (ADSP) gene verification committee (Bellenguez et al. 2022), while four genes, namely, *NEGR1* (rs146486181, OR = 5.7, *P* = 6.4 × 10^− 6^), *EDEM1* (rs378516, OR = 4.7, *P* = 2.1 × 10^− 6^), *ZNF596* (rs35515698, OR = 5.0, *P* = 2.9 × 10^− 6^), and *KCNJ6* (rs148857765, OR = 7.6, *P* = 9.9 × 10^− 6^) have been previously identified in association to AD (Cho et al. 2025; Kunkle et al. 2021; Ni et al. 2018; Zhao et al. 2017). Remarkably, half of the 11 remaining loci is associated with AD-related phenotypes. Specifically, *EDIL3* (rs146486181, OR = 2.4, *P* = 4.7 × 10^− 6^) is associated with *APOE4* over-expression (Watanabe et al. 2023); *GPR39* (rs147748703, OR = 4.2, *P* = 2.7 × 10^− 6^) is a zinc-finger receptor and was found to be localized in the aging human brain with vascular cognitive impairment (Davis et al. 2021; Hershfinkel 2018); *PCDH18* (rs13123787, OR = 1.8, *P* = 2.3 × 10^− 6^) and *SETBP1* (rs139826892, OR = 5.1, *P* = 2.6 × 10^− 6^) were previously reported to be implicated in intellectual disability (Hirabayashi and Yagi 2014; Kasnauskiene et al. 2012; Kohyanagi and Ohama 2023; Leonardi et al. 2020); *PDGFD* (rs12271696, OR = 1.8, *P* = 6.8 × 10^− 6^) plays a role in neurogenesis and synaptogenesis (Sil et al. 2018); and *TPTE* (rs3922752, OR = 0.6, *P* = 6.3 × 10^− 6^) is involved in autism related phenotypes (Rodríguez-Escudero et al. 2011). Among the remaining six genes, *FAT1* (rs72716253, OR = 11.0, *P* = 8.9 × 10^− 6^) and *EVPLL* (rs201518577, OR = 1.9, *P* = 8.6 × 10^− 6^) play roles in cell connections (Fogl et al. 2016); three genes, *VKORC1L1* (rs375214815, OR = 3.3, *P* = 7.8 × 10^− 6^), *STEAP1* (rs200942854, OR = 3.8, *P* = 8.0 × 10^− 6^) and *KCNJ6* (rs148857765, OR = 7.6, *P* = 9.9 × 10^− 6^) are related to micronutrient-dependent functions (Brown et al. 2015; Ohgami et al. 2005); and *PPIAL4G* (rs74992572, OR = 2.3, *P* = 2.0 × 10^− 6^) regulates protein folding (Brown et al. 2015). Of the 15 genomic loci, only *TPTE*’s lead-SNP exhibits a protective effect and common effect allele frequency (EAF) of 0.37, while lead-SNPs in the other genes demonstrate large risk effects and moderate to low EAFs (Table 1).

**Fig. 2:**
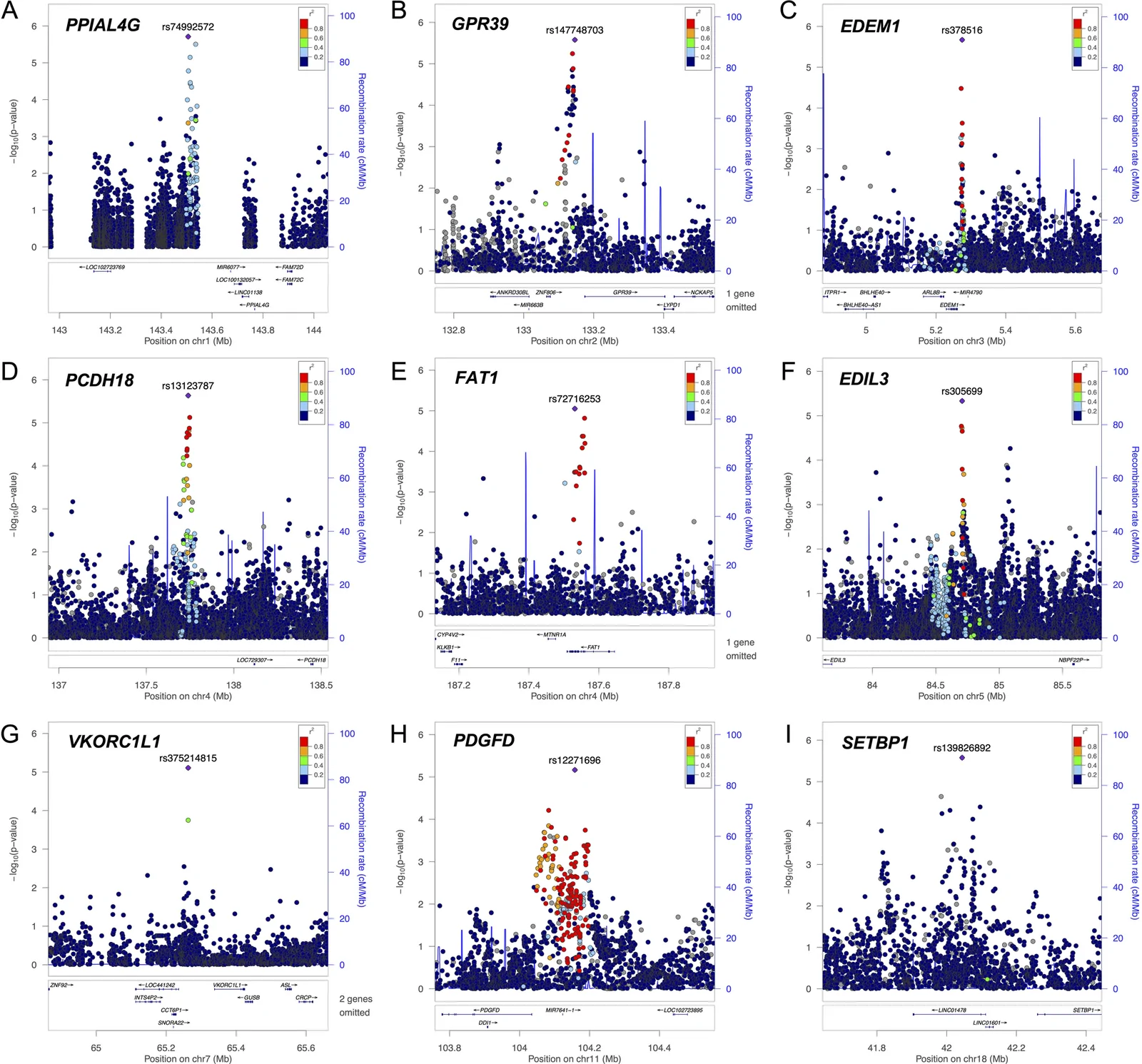
Regional association plots of detected genomic loci associated with cognitive impairment in the Chinese population. Locus-zoom plots demonstrate clear clustering of signals around the lead SNPs of selected loci. All 15 loci detected are illustrated in Figure S4. The color gradient indicates linkage disequilibrium (LD, *r*^*2*^) levels of each SNP relative to the lead SNP (purple diamond) within a given locus. For the lead SNP rs375214815 (panel **G**), which is absent in the 1000 Genomes East Asian LD reference panel, the in-sample LD was estimated using the Zhejiang GWAS samples

Next, we imputed the 15 genomic loci and utilized locus-zoom plots to further examine the evidence of their association. Nine of these genomic loci showed clearly elevated signals clustering surrounding the lead SNPs, including *PPIAL4G*, *GPR39*, *EDEM1*, *PCDH18*, *FAT1*, *EDIL3*, *VKORC1L1*, *PDGFD* and *SETBP1* (Fig. 2, Fig S4).

### Replication studies

**Fig. 3:**
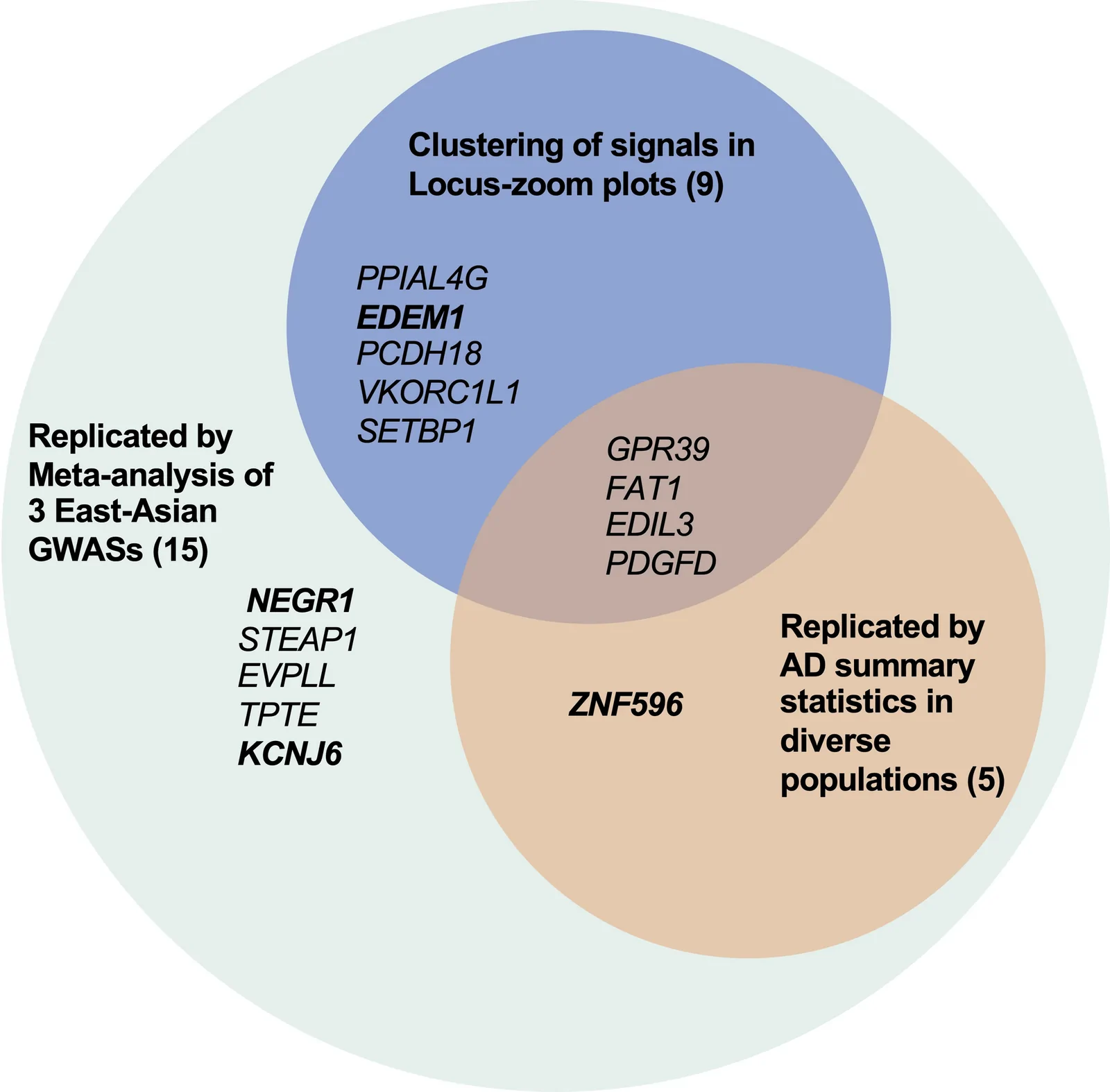
Venn diagram illustrating the replication outcomes for the 15 detected genetic loci associated with cognitive impairment. Among the 15 genes found, five genes, *GPR39*, *FAT1*, *EDIL3*, *ZNF596*, and *PDGFD*, were replicated in both replication studies. Four genes (*GPR39*, *FAT1*, *EDIL3*, and *PDGFD*) also displayed a clustering of signals in the locus-zoom plots. The corresponding loci are detailed in Tables 1, 2 and 3. Genes in boldface have been previously reported to be associated with AD

Two replication studies were conducted to further investigate the genomic loci identified from the Zhejiang (ZJ) GWAS data. A challenge was expected as the detected loci in the primary data predominantly exhibit low to moderate allele frequencies. The first replication study involved a meta-analysis of GWASs across three East Asian populations. In addition to the in-house data, we calculated summary statistics for AD-by-proxy in the UKBB East Asian population and extracted summary statistics from a GWAS of AD in the Japanese population (JP GWAS) (Methods). We calculated the meta-analysis *p*-value (meta-*P*) of all imputed variants within the 15 genomic loci. All 15 loci contained variants with consistent effect direction and meta-*P* < 0.05, and 6 loci were significant at a Bonferroni-corrected level (*P* < 0.05/15 = 3.3 × 10^− 3^) (Table 2; Fig. 3). Of interests were the 3 loci where the meta-*P* was more significant than the *p*-values in any individual GWAS. These include *EDIL3* (meta-*P* = 6.6 × 10^− 4^), *SETBP1* (meta-*P* = 2.1 × 10^− 3^) and *TPTE* (meta-*P* = 1.4 × 10^− 5^). In the second replication study, we examined the *P* values of the lead SNPs (Table 1) or SNPs in LD with the lead SNPs using summary statistics from external GWAS of AD in diverse populations (Table 3; Fig. 3). Five loci, *GPR39*, *FAT1*, *EDIL3*, *ZNF596* and *PDGFD*, contained lead SNP or LD-proxy SNPs with a nominally significant *P* value (*P* < 0.05) in the external GWAS data for AD. Of these loci, *ZNF596* contained the LD proxy rs115852274 (*r*^*2*^ = 0.96) of rs35515698 reached Bonferroni significance (*P* < 0.05/15 = 3.3 × 10^− 3^) in IGAP cohort. Overall, five genomic loci found in the primary ZJ GWAS data showed nominal evidence of replication in both validation studies (Fig. 3): *GRP39*, *FAT1*, *EDIL3*, *ZNF596*, and *PDGFD*, among which four genes (*GRP39*, *FAT1*, *EDIL3*, and *PDGFD*) also demonstrated convincing signals in the locus-zoom plots.

### *APOE* association to cognitive impairment in the Chinese population

Next, we investigated the association of *APOE* with cognitive impairment in the Chinese population by genotyping of its two major variants, rs429358 and rs7412, in 1,040 subjects. Genotyping data were successfully obtained for 1,038 individuals. Using the ɛ3/ɛ3 genotype as the reference group (*N* = 688), the effects of the other genotypes are: ɛ4/ɛ4 (OR = 1.87, *N* = 6, *P* = 0.47), ɛ3/ɛ4 (OR = 1.18, *N* = 172, *P* = 0.37), and ɛ2/ɛ2 (OR = 0.47, *N* = 6, *P* = 0.49) (Table 4, Fig S5). Although the *P*-values were not statistically significant, the observed risk effect of ɛ4 and the protective effect of ɛ2 are consistent with their reported effects on AD in the European population.

### Polygenic risk score analysis

To understand the combined effect of the detected SNPs, we constructed a polygenic risk score (PRS) using the 19 SNPs showing suggestive significance to perform an in-sample evaluation. This PRS accounted for 28% of the observed variance (Nagelkerke’s pseudo *R*^*2*^) in the Zhejiang GWAS data. The OR of PRS and cognitive impairment is 3.38 with *p*-value = 2.5 × 10^− 36^, evaluated by logistic regression in the Zhejiang data. Additionally, individuals in the highest decile of PRS had an OR of 44.5 (95% CI: 20.3–106.1), compared to those in the lowest decile (Fig S6 and Table S2). It should be noted that as the same sample was used to select SNPs, estimate their effect sizes, and evaluate the PRS, the ORs and pseudo *R*^*2*^ values are subject to upward bias and should not be interpreted as the predictive performance of PRS.

## Discussion

In this study, we collected samples and conducted whole genome sequencing of 1,040 individuals in a longitudinal elderly cohort in Zhejiang, China. Through genome-wide association analysis, we found 15 genomic loci with suggestive level of significance linked to dementia in the Chinese population. Eleven of these loci are novel to AD or cognitive impairment traits. Four of them, *GRP39*, *FAT1*, *EDIL3* and *PDGFD*, were further supported by locus-zoom analysis and replication studies. In the following sections, we review biological functions of the detected loci, report the results of their enrichment analyses, and discuss the genetic heterogeneity of dementia and AD across diverse populations. These biological inferences should be regarded as speculative interpretations of suggestive association signals detected.

### Genes involved in the formation of amyloid-β and neurofibrillary tangles

**Fig. 4:**
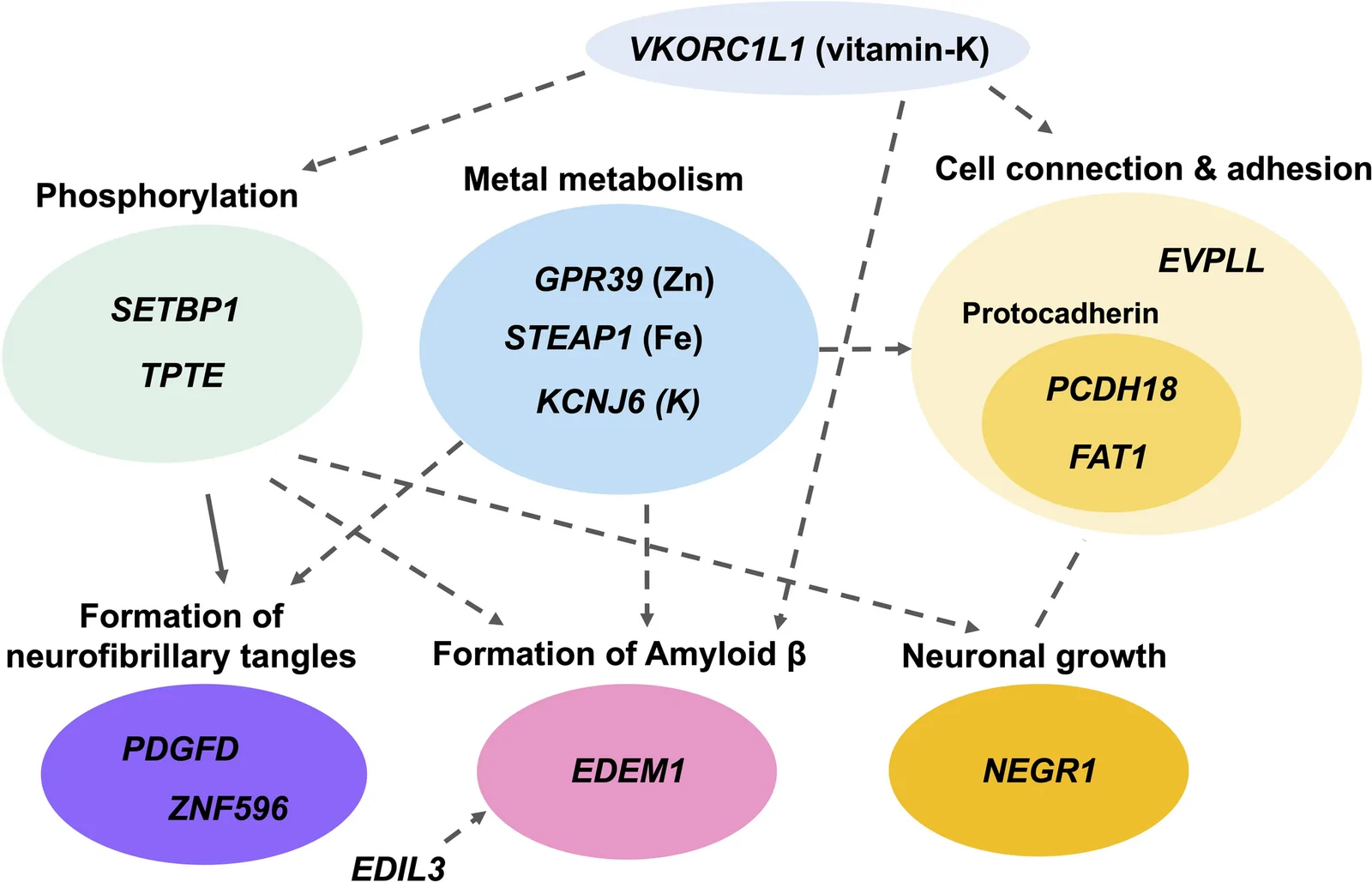
Biological functions of the detected loci from the Chinese population. Fourteen out of the 15 genomic loci detected are implicated in biological processes closely tied to neuronal functions and brain health

Four susceptible loci, *EDEM1*, *ZNF596*, *PDGFD* and *EDIL3*, are involved in the formation of amyloid plaques and neurofibrillary tangles, the two main histopathological signatures of AD (Bloom 2014) (Fig. 4). The *EDEM1* (3p26.1) functions in Endoplasmic Reticulum-associated protein degradation (Hosokawa et al. 2001) and is an important regulator of amyloid-beta precursor protein (APP) metabolism (Nowakowska-Gołacka et al. 2021). *ZNF596* (8p23.3) encodes zinc-finger protein which hypermethylation is associated with the increase of neurofibrillary tangles in human postmortem AD brain (Cassandri et al. 2017; Zhao et al. 2017). The *PDGFD* (or *PDFG-D*) (11q22.3) is a member of platelet-derived growth factor (*PDGF*) family (Sil et al. 2018), whose other members are found to play roles in the mechanisms of neurofibrillary pathology in AD (Masliah et al. 1995) and regulates APP (Masliah et al. 1995). The *EDIL3* (other name *DEL1*) (5q14.3) is a glycoprotein secreted by endothelial cells that interferes leukocyte-endothelial interactions (Choi et al. 2008). Elevated levels in *EDIL3* have been associated to dendritic spine reduction in neuronal cultures and are found in amyloid plaques of postmortem AD brains carrying *APOE4* risk allele (Watanabe et al. 2023).

### Genes functioning in neuronal growth and cell-cell adhesions

Four genes, *NEGR1*, *PCDH18*, *FAT1* and *EVPLL*, play key roles in neuronal growth and cell connections (Fig. 4). The *NEGR1* (1p31.1) is Neuronal Growth Regulator 1, which regulates synapse formation and promotes neurite outgrowth (Hashimoto et al. 2008; Sanz et al. 2015), and was previously found in association to cognitive functions (Davies et al. 2018; Lam et al. 2017; Lee et al. 2018a, b), AD (Kunkle et al. 2019; Sherva et al. 2020), and major depressive disorder (MDD) in the European population (Hyde et al. 2016). Two other genes, *PCDH18* (4q28.3) and *FAT1* (4q35.2), both belong to the protocadherin family within the cadherin superfamily, which regulates calcium-dependent cell adhesion, signal transduction, and synaptic plasticity (László and Lele 2022; Tang et al. 1998) (Fig. 4). Previously, a single deletion in the *PCDH18* was associated with intellectual disability (Kasnauskiene et al. 2012). *FAT1* was found to control neurogenesis mediated through hippo signaling pathway (Ahmed et al. 2015). Lastly, *EVPLL* (17p11.2) is an Envoplakin-Like protein that functions in forming cell junctions and cytoskeletal frameworks (Fogl et al. 2016).

### Protein phosphorylation genes

Notably, two of the susceptible loci, *SETBP1* and *TPTE*, are involved in the regulation of phosphatase. It is well established that hyperphosphorylation of tau contributes to neurofibrillary degeneration in AD (Grundke-Iqbal et al. 1986; Lee et al. 1991). *SETBP1* (18q12.3) is an inhibitor of protein phosphatase 2 A (*PP2A*) (Kohyanagi and Ohama 2023), which accounts for ~ 70% of total tau activity in human brain and is negatively correlated to tau phosphorylation in AD brain (Liu et al. 2005; Sontag and Sontag 2014). Previously, *SETBP1* was identified to be associated with intellectual disability (Leonardi et al. 2020; Taşkıran et al. 2021). While *TPTE* (21p11.2) is a transmembrane phosphatase belonging to the *PTEN* family (Tapparel et al. 2003) that in addition to being a tumor suppressor gene, controls neuronal growth (Griffin et al. 2005; Knafo and Esteban 2017). Recent studies found that *PTEN* rescued normal synaptic function and cognition in cellular and animal models of AD (Knafo et al. 2016).

### Genes functions in metal homeostasis in brain: zinc, iron and potassium

Interestingly, we detected three genes involved in metal homeostasis that may play a role in AD: *GPR39* for Zinc, *STEAP1* for iron, and *KCNJ6* for Potassium. Proper metal trafficking is crucial for the normal functioning of the central nervous system (Que et al. 2008). Growing evidence has indicated the pathological role of metal dysregulation in the development of AD, through inducing oxidative stress, synaptic impairment, and the toxic accumulations of amyloid β plaques and neurofibrillary tau tangles (Ayton et al. 2013; Das et al. 2021). Among the three genes, *GPR39* (2q21.2) acts as a zinc-sensing receptor in brain (Hershfinkel 2018), and is involved in regulating the transport of sodium (Na^+^), potassium (K^+^), and chloride (Cl^−^) that are essential for neuron transmission (Ganay et al. 2015; Rychlik and Mlyniec 2020). Over concentration of zinc is found to induce the formation of Aβ amyloid (Bush et al. 1994). *STEAP1* (7q21.13) is suggested to be an iron channel or transporter protein in cell adhesion (Gomes et al. 2012), playing roles in intracellular communications (Challita-Eid et al. 2007). Abnormally high ferritin levels were found in senile plagues and neurofibrillary tangles of AD brain (Connor et al. 1992; Good et al. 1992), and iron-chelation therapies have shown promise in slowing dementia progression in clinical trials (Schreiner and Schreiner 2023). Notably, we identified *KCNJ6* (21q22.13), a G-protein rectifying potassium channels. A recent study reported *KCNJ6* as a novel East Asian-specific AD-associated functional gene, which shows high expression in neuronal cell types and increased expression with AD pseudo-progression (Cho et al. 2025). This gene belongs to the same family as *KCHJ15* that has previously been associated with AD in the Chinese population (Zhou et al. 2018). Potassium channels are vital for regulating nerve impulses, neuronal balances, and membrane excitability (Villa et al. 2020). Dysfunction in these channels has been detected in the platelets and fibroblast of AD patients (de Silva et al. 1998; Etcheberrigaray et al. 1993).

### Genes functions in vitamin K maintenance

We detected *VKORC1L1* (7q11.21), a protein encoding an enzyme that supports vitamin K-mediated intracellular antioxidation critical to cell survival (Westhofen et al. 2011). The role of vitamin K in neurodegeneration is an emerging area of research (Alisi et al. 2019; Diachenko et al. 2024). Higher level of vitamin K was associated with lower risk of dementia in both elderly people and postmortem AD brain (Booth et al. 2022; Kiely et al. 2020); and vitamin K_3_ was shown to reduce amyloid cytotoxicity by shifting amyloid aggregation towards nontoxic aggregates (Alam et al. 2016).

Overall, fourteen out of the 15 genomic loci detected are implicated in biological processes closely tied to neuronal functions and brain health (Fig. 4). These include the formation of amyloid β (*EDEM1*) and neurofibrillary tangles (*PDGFD*, *ZNF596*), neuronal growth (*NEGR1*), cell connections (*EVPLL*) through cadherin regulations (*PCDH18*, *FAT1*), phosphatase regulations (*SETBP1*, *TPTE*), metal metabolism (*GPR39*, *STEAP1*, *KCNJ6*) and vitamin *K* maintenance (*VKORC1L1*). These biological processes highlight new avenues for developing AD therapeutics and warrant further experimental research.

### Enrichment of cognitive impairment association signals in tissue types and pathways

We evaluated the tissue types enriched with the genetic signals detected in the Zhejiang GWAS data using two methods. First, we applied LD score regression to specifically expressed genes (LDSC-SEG) in 13 brain tissues from GTEx database (Finucane et al. 2018). Analysis suggested that our SNP-based association signals were most strongly implicated in differentially expressed genes in brain’s putamen (*p*-value = 0.008, Table S3). Next, we examined the relationship between gene-based signals and gene expression of 30 general tissue types in the GTEx data by MAGMA (de Leeuw et al. 2015) (Methods). The result indicated that the association signals of cognitive impairment were most enriched in pituitary gland (Table S4, Fig S7). Both putamen and pituitary are implicated in AD pathology. The putamen is involved in motor planning, motivation, emotional regulation, language processing, and memory functions (Kumar et al. 2011). It was shown that degeneration in the putamen and thalamus contributed to cognitive decline in AD (de Jong et al. 2008), while disturbance in pituitary gland, a part of the neuroendocrine system, would result in elevated cortisol levels, cerebrospinal fluid (CSF) cortisol and mineralocorticoid receptors, all of which are associated with AD (Gil-Bea et al. 2010; Pomara et al. 2003), *APOE4* (Gil-Bea et al. 2010), and dementia (Raskind et al. 1982). We then expanded our analysis to pathway-based gene-sets. Out of the 17,004 curated gene sets and gene ontology terms from the MSigDB database (Liberzon et al. 2015; Subramanian et al. 2005), the response to selenium ion showed a significant association with the gene-based signals in the Zhejiang data (Bonferroni-adjusted *p*-value = 7.7 × 10^− 4^). Selenium is essential for brain development (Schweizer and Fabiano 2022), and its deficiency has been linked to an increased risk of cognitive decline (Rayman 2012) and AD (Gwon et al. 2010; Zhang and Song 2021).

### Genetic heterogeneity of AD and cognitive impairment in Chinese and European populations

Finally, we compared the AD and cognitive impairment association signals detected in the Zhejiang GWAS data with those from other populations. The effect alleles of the lead-SNPs in the Zhejiang data were either absent, extremely rare, or common in European and African populations, where they did not show suggestive association signals (Table S5). Conversely, we examined the 75 confirmed AD susceptible SNPs from the European population (Bellenguez et al. 2022) in the Zhejiang dataset. Similarly, after imputation and quality control, these SNPs were either absent, extremely rare, or common in the Zhejiang dataset. Out of the 35 European SNPs that are available in the Zhejiang data, none showed suggestive association signals in our Chinese cohort (Table S6). These cross-population analysis revealed strong genetic heterogeneity underlying AD and cognitive impairment traits, highlighting the importance of conducting GWAS of complex disorders across diverse populations.

### *APOE* association to cognitive impairment

Although *APOE* is well-established as the strongest genetic risk factor for AD, its impact on cognitive performance has been inconsistent (Rahman et al. 2024), particularly in its association to cognitive impairment in the Chinese population (Cong et al. 2023; Jiang et al. 2017; Jin et al. 2021; Zhang et al. 2022). Notably, the *APOE* ε4 allele frequency varies across different ethnic populations. The *APOE* ɛ4 allele frequency in our cohort was 18%, which in comparable to that reported in other Chinese population study (Cong et al. 2023), but considerably lower than in other populations (Northern European 25%, Australis 26%, Oceania 37%, and Central Africa 40%) (Huebbe and Rimbach 2017). In our analysis, the association signals at the *APOE* gene did not reach statistical significance. The lack of significance might be due to phenotype heterogeneity within the study cohort. As the cases were categorized using MMSE scores, the case group encompasses a spectrum of cognitive impairment and might be enriched with neurodegenerative conditions from non-AD etiologies (Knopman et al. 2021; Liu et al. 2024), diluting the effect of *APOE* locus on AD-related cognitive decline. Alternatively, the absence of a significant *APOE* signal may reflect limited statistical power due to sample size constraints in detecting population-specific genetic effects.

### Assessment of cognitive impairment

The MMSE is a widely used 30-point questionnaire as a brief screening tool for providing an overall measure of cognitive impairment (Folstein et al. 1975). However, its accuracy for MCI conversion to dementia varies across studies (Arevalo-Rodriguez et al. 2021). In addition, the MMSE score is highly dependent on educational level. In this study, we applied education-adjusted cut-off scores for cognitive impairment from the Chinese version of the MMSE (Zhang et al. 1990), which are commonly used in Chinese epidemiological studies to define dementia. The Chinese version of the MMSE has shown reasonably good performance in screening for dementia, with reported sensitivity and specificity of 85.2% and 92.7%, respectively (Zhang et al. 1990). Clinical diagnosis of cognitive impairment requires a more comprehensive neuropsychological assessment covering both global and domain-specific cognition, which was not feasible in our cohort. This limitation introduces potential measurement error, which could dilute the strength of association signals in our GWAS analysis.

### Limitations

This study has several limitations. The first one is its small sample size which limits power of the association tests and increases the probability of making the type I error, therefore, variants with moderate effect size were not detected and *p*-value of the top SNPs did not reach genome-wide significance level. The relatively large effect size observed for some of the detected loci may reflect the winner’s curse in small sample GWAS studies. These effect sizes might be inflated and shall be interpreted with caution. Further studies in larger samples are required to provide more accurate effect estimates. We also estimated that the average statistical power to detect the 11 novel loci at a type I error rate of 1 × 10^− 5^ was 53.5% (SD 18.2%). This limitation is supplemented with evidence of association signals from the regional Manhattan plot including imputed variants, external replication studies, and the marker’s direct biological implications in the etiology of brain disorders. Secondly, due to the large population stratification between the Chinese and European, many of the detected SNPs with low frequency in this study are not present in the existing GWASs of the European population (and vice versa), hence, we adopted a replication by high LD approach, which may also invite false positives, whereas the ultimate solution for replication study is to have more independent GWAS studies of the Chinese population for dementia or AD. Thirdly, the cases in our study are classified by the MMSE cutoff values. Therefore, our cases included subjects at various stages of cognitive impairment, introducing heterogeneity of the case group, which could have diluted the strength of association signals in the GWAS analysis.

## Conclusions

In conclusion, through well-designed sampling, sequencing and genome-wide association analysis, we have addressed the gap in identifying genetic association signals for cognitive impairment in Chinese population. This study provides the largest set of association signals with putative biological relevance to cognitive functions in the Chinese population. The biological interpretations presented in this study are speculative and require independent validation. Given the limited number of high-quality GWAS on AD or cognitive impairment in the Chinese population and large population heterogeneity, we recommend focusing on the robust evidence presented in the primary data, locus-zoom analysis, and biological functions, rather than relying exclusively on the existing replication results. We urge increased efforts in sample collection and data sharing for GWAS studies to enhance understanding of the genetic foundations of cognitive impairment and AD in the Chinese population.

**Table 1:** Genomic loci reaching suggestive significance in the Zhejiang GWAS of cognitive impairment

Locus #	Gene^1^	Chr: position (GRCh37)	Lead variant	Effect allele (EA)	EAF	OR	*P*-value	Previous disease association or function^1^
1	*NEGR1*	1:72581185	rs146486181	G	0.01	5.7	6.4 × 10^− 6^	AD, depression
2	*PPIAL4G*	1:143506049	rs74992572	C	0.08	2.3	2.0 × 10^− 6^	protein folding
3	*GPR39*	2:133146166	rs147748703	T	0.02	4.2	2.7 × 10^− 6^	cognitive impairment, zinc-sensing receptor
4	*EDEM1*	3:5274836	rs378516	C	0.02	4.7	2.1 × 10^− 6^	AD
5	*PCDH18*	4:137739407	rs13123787	A	0.12	1.8	2.3 × 10^− 6^	brain development
6	*FAT1*	4:187531497	rs72716253	C	0.01	11.0	8.9 × 10^− 6^	calcium-dependent adhesion
7	*EDIL3*	5:84707550	rs305699	T	0.05	2.4	4.7 × 10^− 6^	*APOE4* association
8	*VKORC1L1*	7:65262807	rs375214815	C	0.03	3.3	7.8 × 10^− 6^	vitamin K metabolism
9	*STEAP1*	7:89684570	rs200942854	TA	0.02	3.8	8.0 × 10^− 6^	iron deficiency anemia
10	*ZNF596*	8:261775	rs35515698	G	0.01	5.0	2.9 × 10^− 6^	AD
11	*PDGFD*	11:104158065	rs12271696	T	0.10	1.8	6.8 × 10^− 6^	central nervous system
12	*EVPLL*	17:18327505	rs201518577	A	0.12	1.9	8.6 × 10^− 6^	envoplakin protein, cell junctions
13	*SETBP1*	18:42044281	rs139826892	T	0.02	5.1	2.6 × 10^− 6^	intellectual disability
14	*TPTE*	21:10163675	rs3922752	G	0.37	0.6	6.3 × 10^− 6^	autism
15	*KCNJ6*	21:39143300	rs148857765	A	0.01	7.6	9.9 × 10^− 6^	AD, potassium channel

^1^Distance to genes and the complete references of disease association are reported in Table S1

*EAF* Effect allele frequency, *OR* odds ratio

**Table 2:** Meta-analysis of three GWAS datasets of East Asian population-Replication study 1

Locus #	Nearest gene	SNP in meta-analysis	*r* ^2^	Distance (kb)	EA	EAF (ZJ)	EAF (UKBB-EA)	EAF (JP)	Beta (ZJ)	Beta (UKBB-EA)	Beta (JP)	*P* (ZJ)	*P* (UKB)	*P* (JP)	Meta-*P*	I^2^	*P*_het_
1	***NEGR1***	**rs2220253**	**0.01**	16	T	0.35	0.5	0.52	**0.04**	**0.1**	**0.09**	**0.64**	**0.44**	**6.0 × 10** ^**− 3**^	**4.4 × 10** ^**− 3**^	**0**	**0.9**
2	*PPIAL4G*	rs78644440	-	29	T	0.08	0.02	-	0.81	0.42	-	3.1 × 10^− 6^	0.36	-	4.6 × 10^− 6^	0	0.4
3	***GPR39***	**rs2315381**	**0.02**	**9**	**C**	**0.2**	**0.33**	**0.26**	**0.17**	**0.09**	**0.05**	**0.12**	**0.45**	**0.16**	**0.05**	**0**	**0.6**
4	***EDEM1***	**rs62253635**	**0.03**	107	T	0.14	0.31	0.25	**0.16**	**0.17**	**0.09**	**0.21**	**0.19**	**0.02**	**4.3 × 10** ^**− 3**^	**0**	**0.7**
5	***PCDH18***	**rs79682227**	**0.01**	**164**	**G**	**0.04**	**0.06**	**0.04**	**0.13**	**0.17**	**0.19**	**0.53**	**0.48**	**0.03**	**0.02**	**0**	**1**
6	***FAT1***	**rs11938564**	**0**	**319**	**G**	**0.1**	**0.12**	**0.13**	**0.13**	**0.29**	**0.11**	**0.38**	**0.08**	**0.03**	**6.1 × 10** ^**− 3**^	**0**	**0.6**
7	***EDIL3***	**rs67953397**	**0.002**	**299**	**G**	**0.29**	**0.34**	**0.31**	**0.06**	**0.17**	**0.11**	**0.52**	**0.14**	**2.0 × 10** ^**− 3**^	**6.6 × 10** ^**− 4**^	**0**	**0.8**
8	***VKORC1L1***	**rs59933587**	**-**	**240**	**A**	**0.02**	**0.01**	**0.02**	**0.48**	**0.17**	**0.25**	**0.06**	**0.81**	**0.05**	**0.01**	**0**	**0.7**
9	***STEAP1***	**rs137906860**	**-**	**414**	**G**	**0.01**	**0.002**	**0.02**	**0.56**	**0.31**	**0.29**	**0.16**	**0.77**	**0.03**	**0.01**	**0**	**0.8**
10	***ZNF596***	**rs75600271**	**0.001**	**67**	**C**	**0.01**	**0.01**	**0.02**	**0.73**	**0.50**	**0.20**	**0.06**	**0.37**	**0.15**	**0.03**	**0**	**0.4**
11	*PDGFD*	rs10895652	0.03	43	C	0.26	0.32	0.42	0.27	0.08	0.05	5.5 × 10^− 3^	0.53	0.10	0.01	54.1	0.1
12	*EVPLL*	rs201518577	1	0	A	0.12	0.005	-	0.65	0.36	-	8.6 × 10^− 6^	0.64	-	1.1 × 10^− 5^	0	0.7
13	***SETBP1***	**rs73479557**	**-**	**0.81**	**G**	**0.10**	**0.11**	**0.12**	**0.44**	**0.04**	**0.12**	**3.2 × 10** ^**− 3**^	**0.82**	**0.02**	**2.1 × 10** ^**− 3**^	**55.3**	**0.1**
14	***TPTE***	**rs377252712**	**-**	**336**	**C**	**0.12**	**0.03**	**-**	**−0.72**	**−0.71**	**-**	**1.7 × 10** ^**− 5**^	**0.35**	**-**	**1.4 × 10** ^**− 5**^	**0**	**1**
15	*KCNJ6*	rs148857765	1	0	A	0.01	0.002	-	2.02	0.64	-	9.9 × 10^− 6^	0.57	-	1.9 × 10^− 4^	19.7	0.3

Meta-analysis using the summary statistics of three GWAS datasets of the East Asian population, including the Zhejiang GWAS of the Chinese population (ZJ), AD-proxy in the UK Biobank East Asian Population (UKBB-EA), and GWAS of AD in the Japanese population (JP). Of the 15 detected loci in the ZJ GWAS data, 6 have meta-analysis *P*-values (meta-*P*) < 0.05/15 = 3.3 × 10^− 3^. Boldface: the loci with more significant meta-*P* than its smallest *P*-values in individual GWAS. The number of variants in the JP GWAS is less than one-tenth of those in the other two datasets. As a result, some variants are only present in two of the datasets

*I*^2^ heterogeneity statistics, *P*_Het_ heterogeneity *P-*value (the larger the better)

**Table 3:** Replicated loci of this study by the summary statistics of GWASs of AD in diverse populations-Replication study 2

Nearest gene	SNP^1^	*P*-value	GWAS datasets^2^, Population
*GPR39*	*rs10205251*	0.04	AD-language, EUR
	*rs6430216*	0.04	AD-no domain, EUR
*FAT1*	rs72716253	0.02	UKBB-AD, EAS
	rs72716253	0.01	UKBB-AD, EUR
*EDIL3*	rs305699	0.03	AD-multiple, EUR
*ZNF596*	*rs115852274*	0.002	IGAP, EUR
	*rs73669422*	0.04	AD-AFR
	*rs116083040*	0.01	AD-language, EUR
	*rs7846271*	0.03	AD-memory, EUR
	*rs146752934*	0.02	AD-no domain, EUR
*PDGFD*	rs12271696	0.02	UKBB-AD, EAS

Details of these datasets and their references are reported in Methods

*UKBB-AD* AD summary statistics from the UK Biobank, *IGAP* Stage 1 of the International Genomics of Alzheimer’s Disease Project, *AD-AFR* AD GWAS from African population (AFR), *EAS* East Asian population

^1^Lead SNPs in the 15 detected loci in the ZJ GWAS data, or SNPs in linkage disequilibrium to the lead SNPs (marked in italic)

^2^Four external replication datasets: the National Institute on Aging Alzheimer’s Disease Data (NIAAD) summary statistics of European population (EUR) by different AD subgroups, including AD with isolated substantial relative language impairment (AD-Language), with isolated substantial relative memory impairment (AD-memory), with multiple domains with substantial relative impairments (AD-multiple), and with no domain of a substantial relative impairment (AD-no domain)

**Table 4:** *APOE* association to cognitive impairment in the Zhejiang cohort

APOE genotype	Total (*N* = 1,038)	Control (*N* = 663)	Case (*N* = 375)	OR [95% CI]	*P*-value
ɛ3/ɛ3^1^	688 (66.3%)	449 (65.3%)	239 (34.7%)	1	-
ɛ2/ɛ2	6 (0.6%)	5 (83.3%)	1 (16.7%)	0.47 [0.05, 4.14]	0.49
ɛ2/ɛ3	154 (14.8%)	93 (60.4%)	61 (39.6%)	1.31 [0.90, 1.91]	0.16
ɛ2/ɛ4	12 (1.2%)	9 (75.0%)	3 (25.0%)	0.56 [0.15, 2.15]	0.40
ɛ3/ɛ4	172 (16.6%)	104 (60.5%)	68 (39.5%)	1.18 [0.82, 1.69]	0.37
ɛ4/ɛ4	6 (0.6%)	3 (50.0%)	3 (50.0%)	1.87 [0.34, 10.16]	0.47

Calculated using the generalized linear mixed model adjusted for age, gender, education level, smoking status, drinking status, and population structure (genetic relationship matrix)

*OR* odds ratios, *CI* confidence interval

^1^The reference group

## Supplementary Information

Below is the link to the electronic supplementary material.

Supplementary Material 1

## Data Availability

The datasets supporting the conclusions of this article are available in the main text and the supplementary materials.
