# Supplementary material for: A genome-wide association study of cognitive impairment in a Chinese cohort of 1,040 individuals
Source: Hum Genet. 2026 Oct 17;145(1):87. doi: 10.1007/s00439-026-02887-6 (PMC13675299; doi:10.1007/s00439-026-02887-6)
Supplement: Supplementary file 1 [file 439_2026_2887_MOESM1_ESM.docx]

**A genome-wide association study of cognitive impairment in a Chinese cohort of 1,040 individuals**

- **Supplementary Materials**

# Figure S1. Histogram of MMSE score of cases by education group.

**Legend**: The education-adjusted cutoff scores for cognitive impairment are <17 for illiteracy, <20 for primary education, and <24 for secondary education or higher in Chinese population.

# Figure S2. Quantile-quantile plot for cognitive impairment.

**Legend:** Quantile-quantile plot of *p*-values from the primary dataset.

# Figure S3. PCA projections of the subjects from 1000 Genomes reference populations and in-house cohort.

**Legend:** Each dot in the graph represents an individual and is colored by corresponding ancestry. Red is used for African populations, orange for Admixed American populations, blue for East Asian populations, green for European populations, purple for South Asian populations, and black for in-house WGS cohort.

# Figure S4. Locus-zoom plots for genomic loci regions reaching suggestive level of genome-wide significance for cognitive impairment.

**Legend:** The color of the points indicates the linkage disequilibrium (LD, r^2^) with the lead SNP (purple diamond) based on the 1000 Genomes Project East Asian reference information. Grey points represent the lead SNP or nearby SNPs are not in the reference panel, therefore LD values between the lead SNP and nearby SNPs are unavailable. For the lead SNPs (rs74992572, rs375214815, rs200942854, rs3922752) not present in the 1000 Genomes East Asian LD reference panel, the in-sample LD estimated from the Zhejiang GWAS samples was used.

# Figure S5. Association of *APOE* genotypes with cognitive impairment risk using a generalized linear mixed model.

**Legend:** Alzheimer’s Disease risk per *APOE* genotype was compared with the APOE ε3/ε3 reference group. Calculated using the generalized linear mixed model adjusted for age, gender, education level, smoking status, drinking status, and population structure (genetic relationship matrix).

# Figure S6. Decile plot with 95% CI for the PRS (P-value threshold of 1×10^-5^).

**Legend:** For each decile, the odds ratio was estimated from logistic regression (error bars indicate 95% confidence interval (CI) of the estimate). The dashed horizontal line indicates an odds ratio of 1. Sample size of each decile is provided in **Table S2**.

# Figure S7. Tissue expression enrichment in GTEx tissue types.

**Legend:** MAGMA gene property enrichment analysis in 30 general tissue types from GTEx V8.

# Table S1. Variants reaching suggestive level of significance (<10^-5^) in the Zhejiang GWAS of cognitive impairment.

| **Locus #** | **Gene** | **Variant** | **Chr:position  (GRCh37)** | **Distance to nearest gene (kb)** | **Effect allele (EA)/non-effect allele** | **EA**  **Frequency** | **OR** | ***P*-value** | **Previous disease association or gene function** | **References** | **Sig in meta-analysis** | **Sig in AD summary statistics** |
| --- | --- | --- | --- | --- | --- | --- | --- | --- | --- | --- | --- | --- |
| **1** | ***NEGR1*** | **rs146486181** | **1:72581185** | **0** | **G/C** | **0.01** | **5.66** | **6.41×10^-6^** | **depression, AD** | **[1, 2]** | **Y** | **N** |
| 2 | *PPIAL4G* | rs66927428 | 1:142658807 | 1,108 | T/C | 0.06 | 2.47 | 3.37×10^-6^ | protein folding | [3] | Y | N |
| 2 | *PPIAL4G* | rs74992572 | 1:143506049 | 261 | C/T | 0.08 | 2.27 | 1.95×10^-6^ |  |  |  |  |
| 2 | *PPIAL4G* | rs79568859 | 1:143513523 | 254 | A/G | 0.08 | 2.21 | 7.10×10^-6^ |  |  |  |  |
| 2 | *PPIAL4G* | rs78644440 | 1:143535456 | 232 | T/G | 0.08 | 2.25 | 3.13×10^-6^ |  |  |  |  |
| **3** | ***GPR39*** | **rs9989761** | **2:133139402** | **35** | **G/T** | **0.03** | **2.79** | **5.69×10^-6^** | **cognitive impairment,**  **zinc-sensing receptor** | **[4, 5]** | **Y** | **Y** |
| **3** | ***GPR39*** | **rs147748703** | **2:133146166** | **28** | **T/G** | **0.02** | **4.19** | **2.65×10^-6^** |  |  |  |  |
| **4** | ***EDEM1*** | **rs378516** | **3:5274836** | **13** | **C/T** | **0.02** | **4.68** | **2.13×10^-6^** | **AD** | **[6]** | **Y** | **N** |
| **5** | ***PCDH18*** | **rs13123787** | **4:137739407** | **701** | **A/C** | **0.12** | **1.83** | **2.32×10^-6^** | **brain development** | **[7, 8]** | **Y** | **N** |
| 6 | *FAT1* | rs72716253 | 4:187531497 | 0 | C/T | 0.01 | 11.00 | 8.88×10^-6^ | calcium-dependent adhesion | [9] | Y | Y |
| **7** | ***EDIL3*** | **rs305699** | **5:84707550** | **1,027** | **T/C** | **0.05** | **2.35** | **4.65×10^-6^** | ***APOE4* association** | **[10]** | **Y** | **Y** |
| 8 | *VKORC1L1* | rs375214815 | 7:65262807 | 75 | C/T | 0.03 | 3.29 | 7.82×10^-6^ | vitamin K metabolism | [3] | N | N |
| 9 | *STEAP1* | rs200942854 | 7:89684570 | 99 | TA/T | 0.02 | 3.84 | 8.01×10^-6^ | iron deficiency anemia | [11] | Y | N |
| **10** | ***ZNF596*** | **rs35515698** | **8:261775** | **64** | **G/C** | **0.01** | **5.01** | **2.88×10^-6^** | **AD** | **[12]** | **N** | **Y** |
| **11** | ***PDGFD*** | **rs12271696** | **11:104158065** | **123** | **T/G** | **0.10** | **1.81** | **6.83×10^-6^** | **central nervous system** | **[13]** | **Y** | **Y** |
| 12 | *EVPLL* | rs201518577 | 17:18327505 | 35 | A/G | 0.12 | 1.92 | 8.56×10^-6^ | envoplakin protein, cell junctions | [14] | Y | N |
| **13** | ***SETBP1*** | **rs139826892** | **18:42044281** | **216** | **T/G** | **0.02** | **5.07** | **2.64×10^-6^** | **intellectual disability** | **[15, 16]** | **Y** | **N** |
| **14** | ***TPTE*** | **rs3922752** | **21:10163675** | **743** | **G/C** | **0.37** | **0.59** | **6.28×10^-6^** | **autism** | **[17]** | **Y** | **N** |
| **15** | ***KCNJ6*** | **rs148857765** | **21:39143300** | **0** | **A/G** | **0.01** | **7.57** | **9.94×10^-6^** | **AD, potassium channel** | **[3, 18]** | **Y** | **N** |

**Legend:** Variants were annotated with the nearest genes and distance to nearest gene in kb (0, SNP located within gene body). **Boldface:** genes previously identified to be associated with AD or AD related phenotypes. Y, yes; N, no.

**References**

1.	G. Maccarrone *et al.*, Psychiatric patient stratification using biosignatures based on cerebrospinal fluid protein expression clusters. *J Psychiatr Res* **47**, 1572-1580 (2013).

2.	H. Ni *et al.*, The GWAS Risk Genes for Depression May Be Actively Involved in Alzheimer's Disease. *J Alzheimers Dis* **64**, 1149-1161 (2018).

3.	Gene [Internet]. Bethesda (MD): National Library of Medicine (US), National Center for Biotechnology Information; 2004 – [cited 2025 Feb 05]. Available from: https://www.ncbi.nlm.nih.gov/gene/.

4.	C. M. Davis *et al.*, GPR39 localization in the aging human brain and correlation of expression and polymorphism with vascular cognitive impairment. *Alzheimers Dement (N Y)* **7**, e12214 (2021).

5.	M. Hershfinkel, The Zinc Sensing Receptor, ZnR/GPR39, in Health and Disease. *Int J Mol Sci* **19** (2018).

6.	B. W. Kunkle *et al.*, Novel Alzheimer Disease Risk Loci and Pathways in African American Individuals Using the African Genome Resources Panel: A Meta-analysis. *JAMA Neurol* **78**, 102-113 (2021).

7.	M. Wennström, H. M. Nielsen, Cell adhesion molecules in Alzheimer's disease. *Degener Neurol Neuromuscul Dis* **2**, 65-77 (2012).

8.	J. Kasnauskiene *et al.*, A single gene deletion on 4q28.3: PCDH18--a new candidate gene for intellectual disability? *Eur J Med Genet* **55**, 274-277 (2012).

9.	Z. Peng, Y. Gong, X. Liang, Role of FAT1 in health and disease. *Oncol Lett* **21**, 398 (2021).

10.	H. Watanabe *et al.*, Astrocytic APOE4 genotype-mediated negative impacts on synaptic architecture in human pluripotent stem cell model. *Stem Cell Reports* **18**, 1854-1869 (2023).

11.	R. S. Ohgami *et al.*, Identification of a ferrireductase required for efficient transferrin-dependent iron uptake in erythroid cells. *Nat Genet* **37**, 1264-1269 (2005).

12.	J. Zhao *et al.*, A genome-wide profiling of brain DNA hydroxymethylation in Alzheimer's disease. *Alzheimers Dement* **13**, 674-688 (2017).

13.	S. Sil, P. Periyasamy, A. Thangaraj, E. T. Chivero, S. Buch, PDGF/PDGFR axis in the neural systems. *Mol Aspects Med* **62**, 63-74 (2018).

14.	C. Fogl *et al.*, Mechanism of intermediate filament recognition by plakin repeat domains revealed by envoplakin targeting of vimentin. *Nat Commun* **7**, 10827 (2016).

15.	N. Kohyanagi, T. Ohama, The impact of SETBP1 mutations in neurological diseases and cancer. *Genes Cells* **28**, 629-641 (2023).

16.	E. Leonardi *et al.*, Identification of SETBP1 Mutations by Gene Panel Sequencing in Individuals With Intellectual Disability or With "Developmental and Epileptic Encephalopathy". *Front Neurol* **11**, 593446 (2020).

17.	I. Rodríguez-Escudero *et al.*, A comprehensive functional analysis of PTEN mutations: implications in tumor- and autism-related syndromes. *Hum Mol Genet* **20**, 4132-4142 (2011).

18.	Cho M, Chaudhuri S, Liu S, et al. Functional insight into East Asian-specific genetic risk loci for Alzheimer's disease. *Alzheimers Dement*. 2025;21(2):e14553.

# Table S2. Association of PRS with cognitive impairment by decile in Zhejiang data.

| **Decile** | **Controls** | **Cases** | **OR (95% CI)** | ***P*-value** |
| --- | --- | --- | --- | --- |
| 1 | 91 | 11 | 1 | 1 |
| 2 | 85 | 17 | 1.65 [0.74, 3.83] | 0.23 |
| 3 | 81 | 21 | 2.14 [0.99, 4.87] | 0.06 |
| 4 | 80 | 22 | 2.28 [1.06, 5.14] | 0.04 |
| 5 | 71 | 31 | 3.61 [1.74, 7.98] | 8.5 × 10^-4^ |
| 6 | 74 | 28 | 3.13 [1.50, 6.96] | 3.3 × 10^-3^ |
| 7 | 57 | 45 | 6.53 [3.22, 14.24] | 6.2 × 10^-7^ |
| 8 | 54 | 48 | 7.35 [3.63, 16.03] | 1.1 × 10^-7^ |
| 9 | 41 | 61 | 12.31 [6.07, 26.96] | 3.0 × 10^-11^ |
| 10 | 16 | 86 | 44.47 [20.33, 106.12] | 1.5 × 10^-19^ |

**Legend:** For each decile, the odds ratio was estimated using logistic regression by comparing each of the upper nine PRS deciles with the first decile.

# Table S3. Heritability of cognitive impairment partitioned by brain region gene expression.

| **Brain region** | **Coefficient** | **SE** | ***P*-value** |
| --- | --- | --- | --- |
| Brain_Putamen_(basal_ganglia) | 4.2×10^-7^ | 1.8×10^-7^ | 0.008 |
| Brain_Hypothalamus | 1.5×10^-7^ | 1.7×10^-7^ | 0.19 |
| Brain_Cerebellar_Hemisphere | 1.8×10^-7^ | 2.3×10^-7^ | 0.21 |
| Brain_Cerebellum | 1.5×10^-7^ | 2.6×10^-7^ | 0.29 |
| Brain_Caudate_(basal_ganglia) | 9.8×10^-8^ | 1.8×10^-7^ | 0.29 |
| Brain_Amygdala | 8.6×10^-10^ | 1.8×10^-7^ | 0.50 |
| Brain_Frontal_Cortex_(BA9) | -5.3×10^-10^ | 1.8×10^-7^ | 0.50 |
| Brain_Nucleus_accumbens_(basal_ganglia) | -4.4×10^-9^ | 1.8×10^-7^ | 0.51 |
| Brain_Cortex | -5.9×10^-8^ | 2.1×10^-7^ | 0.61 |
| Brain_Hippocampus | -4.8×10^-8^ | 1.8×10^-7^ | 0.61 |
| Brain_Substantia_nigra | -6.0×10^-8^ | 1.9×10^-7^ | 0.62 |
| Brain_Spinal_cord_(cervical_c-1) | -1.6×10^-7^ | 2.0×10^-7^ | 0.79 |
| Brain_Anterior_cingulate_cortex_(BA24) | -1.8×10^-7^ | 1.9×10^-7^ | 0.83 |

**Legend:** The LD score regression applied to specifically expressed genes (LDSC-SEG) in 13 brain tissues from the GTEx data. SE, Standard Error.

# Table S4. MAGMA Tissue expression enrichment in GTEx tissue types.

| **30 General tissues** | **N genes** | **BETA** | **BETA_STD** | **SE** | **Fold-change^1^** | ***P*-value** |
| --- | --- | --- | --- | --- | --- | --- |
| Pituitary | 16750 | 0.02 | 0.03 | 0.01 | 1.00 | 0.04 |
| Nerve | 16750 | 0.01 | 0.02 | 0.01 | 1.27 | 0.13 |
| Adipose_Tissue | 16750 | 0.01 | 0.03 | 0.01 | 1.04 | 0.14 |
| Blood_Vessel | 16750 | 0.01 | 0.02 | 0.01 | 1.30 | 0.16 |
| Fallopian_Tube | 16750 | 0.01 | 0.03 | 0.01 | 1.11 | 0.16 |
| Colon | 16750 | 0.01 | 0.03 | 0.02 | 1.09 | 0.19 |
| Spleen | 16750 | 0.00 | 0.01 | 0.01 | 3.13 | 0.27 |
| Salivary_Gland | 16750 | 0.01 | 0.01 | 0.01 | 2.33 | 0.28 |
| Esophagus | 16750 | 0.01 | 0.02 | 0.02 | 1.59 | 0.29 |
| Stomach | 16750 | 0.01 | 0.01 | 0.01 | 2.13 | 0.30 |
| Kidney | 16750 | 0.00 | 0.01 | 0.01 | 3.55 | 0.33 |
| Lung | 16750 | 0.00 | 0.00 | 0.01 | 6.51 | 0.41 |
| Uterus | 16750 | 0.00 | 0.00 | 0.01 | 11.20 | 0.46 |
| Ovary | 16750 | 0.00 | 0.00 | 0.01 | 73.52 | 0.49 |
| Skin | 16750 | 0.00 | 0.00 | 0.01 | -26.26 | 0.52 |
| Breast | 16750 | 0.00 | -0.01 | 0.02 | -4.24 | 0.59 |
| Thyroid | 16750 | 0.00 | 0.00 | 0.01 | -6.09 | 0.59 |
| Adrenal_Gland | 16750 | 0.00 | -0.01 | 0.01 | -5.36 | 0.61 |
| Liver | 16750 | 0.00 | 0.00 | 0.01 | -6.45 | 0.64 |
| Brain | 16750 | 0.00 | 0.00 | 0.01 | -5.65 | 0.65 |
| Testis | 16750 | 0.00 | 0.00 | 0.01 | -6.37 | 0.66 |
| Prostate | 16750 | -0.01 | -0.01 | 0.01 | -2.40 | 0.67 |
| Bladder | 16750 | -0.01 | -0.02 | 0.02 | -1.61 | 0.72 |
| Small_Intestine | 16750 | -0.01 | -0.01 | 0.01 | -2.43 | 0.72 |
| Pancreas | 16750 | -0.01 | -0.01 | 0.01 | -2.76 | 0.73 |
| Blood | 16750 | 0.00 | -0.01 | 0.01 | -3.50 | 0.75 |
| Muscle | 16750 | -0.01 | -0.01 | 0.01 | -2.95 | 0.77 |
| Cervix_Uteri | 16750 | -0.01 | -0.02 | 0.01 | -1.27 | 0.80 |
| Heart | 16750 | -0.01 | -0.01 | 0.01 | -1.81 | 0.80 |
| Vagina | 16750 | -0.02 | -0.03 | 0.01 | -0.96 | 0.89 |

**Legend:** MAGMA gene property enrichment analysis in 30 general tissue types from GTEx V8. 1. The fold-change value was calculated by dividing the BETA coefficient of pituitary tissue by the BETA value of other tissues. BETA: the regression coefficient; BETA_STD, Semi-standardized regression coefficient; SE, Standard Error.

# Table S5. Allele frequency and *p*-value of the identified susceptible loci in the Zhejiang GWAs data in other populations in the UK Biobank data.

| **Locus #** | **Gene** | **Lead variant** | **Effect allele (EA)/non-effect allele** | **ZJ GWAS** | | **UKB EAS** | | **UKB EUR** | | **UKB AFR** | | **UKB SAS** | |
| --- | --- | --- | --- | --- | --- | --- | --- | --- | --- | --- | --- | --- | --- |
|  |  |  |  | **EAF** | ***P* value** | **EAF** | ***P-*value** | **EAF** | ***P* value** | **EAF** | ***P-*value** | **EAF** | ***P-*value** |
| 1 | *NEGR1* | rs146486181 | G/C | 0.01 | 6.41 × 10^-6^ | 0.001 | 0.93 | 0.00 | 0.43 | 0.00 | NA | 0.00 | NA |
| 2 | *PPIAL4G* | rs66927428 | T/C | 0.06 | 3.37 × 10^-6^ | 0.01 | 0.77 | 0.07 | 0.78 | 0.04 | 0.33 | 0.04 | 0.16 |
| 2 | *PPIAL4G* | rs74992572 | C/T | 0.08 | 1.95 × 10^-6^ | NA | NA | NA | NA | NA | NA | NA | NA |
| 2 | *PPIAL4G* | rs79568859 | A/G | 0.08 | 7.10 × 10^-6^ | NA | NA | NA | NA | NA | NA | NA | NA |
| 2 | *PPIAL4G* | rs78644440 | T/G | 0.08 | 3.13 × 10^-6^ | 0.02 | 0.36 | 0.16 | 0.77 | 0.31 | 0.21 | 0.09 | 0.69 |
| 3 | *GPR39* | rs9989761 | G/T | 0.03 | 5.69 × 10^-6^ | 0.04 | 0.77 | 0.21 | 0.70 | 0.38 | 0.08 | 0.13 | 0.48 |
| 3 | *GPR39* | rs147748703 | T/G | 0.02 | 2.65 × 10^-6^ | 0.02 | 0.22 | 0.00 | 0.63 | 0.001 | 0.81 | 0.002 | 0.96 |
| 4 | *EDEM1* | rs378516 | C/T | 0.02 | 2.13 × 10^-6^ | 0.05 | 0.19 | 0.12 | 0.98 | 0.28 | 0.54 | 0.09 | 0.80 |
| 5 | *PCDH18* | rs13123787 | A/C | 0.12 | 2.32 × 10^-6^ | 0.16 | 0.43 | 0.31 | 0.43 | 0.04 | 0.75 | 0.24 | 0.85 |
| 6 | *FAT1* | rs72716253 | C/T | 0.01 | 8.88 × 10^-6^ | 0.02 | 0.02 | 0.33 | 0.01 | 0.04 | 0.36 | 0.25 | 0.70 |
| 7 | *EDIL3* | rs305699 | T/C | 0.05 | 4.65 × 10^-6^ | 0.06 | 0.17 | 0.24 | 0.44 | 0.27 | 0.54 | 0.19 | 0.09 |
| 8 | *VKORC1L1* | rs375214815 | C/T | 0.03 | 7.82 × 10^-6^ | NA | NA | NA | NA | NA | NA | NA | NA |
| 9 | *STEAP1* | rs200942854 | TA/T | 0.02 | 8.01 × 10^-6^ | 0.01 | 0.08 | 0.00 | 0.95 | 0.00 | 0.60 | 0.001 | 1.00 |
| 10 | *ZNF596* | rs35515698 | G/C | 0.01 | 2.88 × 10^-6^ | 0.04 | 0.75 | 0.006 | 0.82 | 0.45 | 0.79 | 0.03 | 0.09 |
| 11 | *PDGFD* | rs12271696 | T/G | 0.10 | 6.83 × 10^-6^ | 0.14 | 0.02 | 0.45 | 0.14 | 0.32 | 0.30 | 0.42 | 0.64 |
| 12 | *EVPLL* | rs201518577 | A/G | 0.12 | 8.56 × 10^-6^ | 0.01 | 0.64 | 0.006 | 0.34 | 0.02 | 0.20 | 0.01 | 0.24 |
| 13 | *SETBP1* | rs139826892 | T/G | 0.02 | 2.64 × 10^-6^ | 0.01 | 0.58 | 0.00 | 0.43 | 0.001 | 0.14 | 0.001 | 0.70 |
| 14 | *TPTE* | rs3922752 | G/C | 0.37 | 6.28 × 10^-6^ | NA | NA | NA | NA | NA | NA | NA | NA |
| 15 | *KCNJ6* | rs148857765 | A/G | 0.01 | 9.94 × 10^-6^ | 0.002 | 0.57 | 0.00 | 0.80 | 0.007 | 0.98 | 0.00 | 0.67 |

**Legend:** ZJ, Zhejiang; UKB, UK Biobank; EAS, East Asian; EUR, European; AFR, African; SAS, South Asian; EAF, effect allele frequency; OR, odds ratio.

# Table S6. Allele frequency and p-value of the confirmed susceptible loci for AD in the Zhejiang GWAS data.

| **Gene** | **SNP** | **Effect allele (EA)** | **European** | | | **Chinese**  **(Zhejiang Data)** | | |
| --- | --- | --- | --- | --- | --- | --- | --- | --- |
|  |  |  | **EAF** | **OR** | ***P-*value** | **EAF** | **OR** | ***P-*value** |
| *ADAM17* | rs72777026 | G | 0.14 | 1.06 | 2.72 × 10^-8^ | 0.09 | 0.89 | 0.48 |
| *BIN1* | rs6733839 | T | 0.39 | 1.17 | 6.06 × 10^-118^ | 0.24 | 1.00 | 1.00 |
| *WDR12* | rs139643391 | T | 0.13 | 0.94 | 1.08 × 10^-8^ | 0.01 | 0.26 | 0.02 |
| *CLNK* | rs6846529 | C | 0.28 | 1.07 | 2.20 × 10^-17^ | 0.15 | 1.13 | 0.30 |
| *ANKH* | rs112403360 | A | 0.07 | 1.09 | 2.27 × 10^-9^ | 0.02 | 0.98 | 0.94 |
| *COX7C* | rs62374257 | C | 0.23 | 1.07 | 1.38 × 10^-15^ | 0.17 | 0.89 | 0.30 |
| *UNC5CL* | rs10947943 | A | 0.14 | 0.94 | 1.13 × 10^-9^ | 0.05 | 1.15 | 0.49 |
| *CD2AP* | rs7767350 | T | 0.27 | 1.08 | 7.94 × 10^-22^ | 0.09 | 1.01 | 0.96 |
| *ICA1* | rs10952097 | T | 0.11 | 1.07 | 6.81 × 10^-9^ | 0.05 | 0.72 | 0.13 |
| *TMEM106B* | rs13237518 | A | 0.41 | 0.96 | 4.88 × 10^-11^ | 0.63 | 1.02 | 0.82 |
| *EPDR1* | rs6966331 | T | 0.35 | 0.96 | 4.64 × 10^-10^ | 0.51 | 1.00 | 0.98 |
| *SPDYE3* | rs7384878 | C | 0.31 | 0.92 | 1.06 × 10^-26^ | 0.58 | 0.91 | 0.31 |
| *PTK2B* | rs73223431 | T | 0.37 | 1.07 | 4.03 × 10^-22^ | 0.15 | 1.11 | 0.39 |
| *USP6NL* | rs7912495 | G | 0.46 | 1.06 | 9.74 × 10^-19^ | 0.22 | 0.94 | 0.60 |
| *ANK3* | rs7068231 | T | 0.40 | 0.95 | 3.32 × 10^-13^ | 0.74 | 1.02 | 0.86 |
| *BLNK* | rs6584063 | G | 0.04 | 0.89 | 6.73 × 10^-11^ | 0.01 | 0.68 | 0.48 |
| *PLEKHA1* | rs7908662 | G | 0.47 | 0.96 | 2.59 × 10^-9^ | 0.69 | 0.83 | 0.05 |
| *SPI1* | rs10437655 | A | 0.40 | 1.06 | 5.28 × 10^-14^ | 0.15 | 1.19 | 0.17 |
| *MS4A4A* | rs1582763 | A | 0.37 | 0.91 | 3.74 × 10^-42^ | 0.07 | 0.95 | 0.76 |
| *EED* | rs3851179 | T | 0.36 | 0.90 | 2.95 × 10^-48^ | 0.28 | 0.90 | 0.32 |
| *SORL1* | rs74685827 | G | 0.02 | 1.19 | 2.81 × 10^-11^ | 0.03 | 1.55 | 0.09 |
| *SORL1* | rs11218343 | C | 0.04 | 0.84 | 1.40 × 10^-21^ | 0.09 | 1.12 | 0.49 |
| *TPCN1* | rs6489896 | C | 0.08 | 1.08 | 1.80 × 10^-9^ | 0.10 | 1.03 | 0.82 |
| *FERMT2* | rs17125924 | G | 0.09 | 1.10 | 8.32 × 10^-16^ | 0.10 | 1.05 | 0.71 |
| *IGH gene cluster* | rs10131280 | A | 0.13 | 0.94 | 4.26 × 10^-10^ | 0.12 | 0.75 | 0.05 |
| *SPPL2A* | rs8025980 | G | 0.35 | 0.96 | 1.32 × 10^-8^ | 0.36 | 1.08 | 0.44 |
| *MINDY2* | rs602602 | A | 0.28 | 0.94 | 2.07 × 10^-15^ | 0.51 | 1.10 | 0.31 |
| *SNX1* | rs3848143 | G | 0.22 | 1.05 | 8.41 × 10^-11^ | 0.14 | 0.97 | 0.83 |
| *CTSH* | rs12592898 | A | 0.13 | 0.94 | 4.18 × 10^-9^ | 0.05 | 0.95 | 0.78 |
| *MAF* | rs450674 | C | 0.37 | 0.96 | 3.16 × 10^-8^ | 0.03 | 1.16 | 0.55 |
| *FOXF1* | rs16941239 | A | 0.03 | 1.13 | 1.29 × 10^-8^ | 0.10 | 0.98 | 0.89 |
| *PRDM7* | rs56407236 | A | 0.07 | 1.11 | 6.47 × 10^-15^ | 0.02 | 0.72 | 0.30 |
| *SCIMP* | rs7225151 | A | 0.12 | 1.08 | 4.13 × 10^-13^ | 0.03 | 0.81 | 0.40 |
| *APP* | rs2154481 | C | 0.48 | 0.95 | 1.00 × 10^-12^ | 0.46 | 0.88 | 0.16 |
| *ADAMTS1* | rs2830489 | T | 0.28 | 0.95 | 1.69 × 10^-10^ | 0.01 | 1.61 | 0.27 |

**Legend:** EAF, effect allele frequency; OR, odds ratio.
